# Increased Risk of Retinal Vasculitis in Patients With Systemic Lupus Erythematosus: A Nationwide Population-Based Cohort Study

**DOI:** 10.3389/fmed.2021.739883

**Published:** 2021-09-28

**Authors:** Xiao-Huan Chen, Jia-Cheng Shi, James Cheng-Chung Wei, Hsin-Hua Chen, Han-You Mo

**Affiliations:** ^1^Department of Endocrinology and Rheumatology, The First People's Hospital of Linping District, Hangzhou, China; ^2^Department of Rheumatology, Guilin Medical University, Guilin, China; ^3^Department of Nephrology, Haining People's Hospital, Jiaxing, China; ^4^Department of Endocrinology, Guilin Medical University, Guilin, China; ^5^Institute of Medicine, Chung Shan Medical University Hospital, Taichung, Taiwan; ^6^Department of Allergy, Immunology and Rheumatology, Chung Shan Medical University Hospital, Taichung, Taiwan; ^7^Graduate Institute of Integrated Medicine, China Medical University, Taichung, Taiwan; ^8^Division of Allergy, Immunology and Rheumatology, Taichung Veterans General Hospital, Taichung, Taiwan; ^9^Department of Medical Research, Taichung Veterans General Hospital, Taichung, Taiwan; ^10^Department of Industrial Engineering and Enterprise Information, Tunghai University, Taichung, Taiwan; ^11^School of Medicine, China Medical University, Taichung, Taiwan; ^12^Institute of Biomedical Science and Rong Hsing Research Centre for Translational Medicine, Chung Hsing University, Taichung, Taiwan; ^13^Department of Rheumatology, The Affiliated Hospital of Guilin Medical University, Guilin, China

**Keywords:** retinal vasculitis, systemic lupus erythematosus, epidemiology, cohort study, database

## Abstract

**Objectives:** To evaluate the relationship between systemic lupus erythematosus (SLE) and the risk of retinal vasculitis (RV) using a population-based database.

**Methods:** Using the 1997–2013 Taiwanese National Health Insurance Database, we identified newly diagnosed SLE patients between 2001 and 2012 as the SLE group. We matched the SLE group with non-SLE individuals selected from a representative one million sample of the population in a 1:20 ratio for age, sex, and the year of the index date. After adjusting for potential confounders, including urbanization of the patient's residence, the level of the payroll-related insured amount, and selected comorbidities, we examined the association between SLE and the risk of RV using the Cox proportional hazard model shown as hazard ratios (HRs) with 95% confidence intervals (CIs). Sensitivity analyses were conducted using various definitions of RV.

**Results:** We included 11,586 patients with SLE and 231,720 matched non-SLE individuals. The mean age of the study participants was 36.7 ± 16.9 years, and the female-to-male ratio was 6.8:1. The incidence rates of RV were 56.39 cases per 100,000 person-years and 2.45 cases per 100,000 person-years, respectively. After adjusting for potential confounders, the incidence rate of RV in the SLE cohort was 22.99 times higher than that in the non-SLE cohort (56.39 vs. 2.45 per 100,000 person-years). The adjusted HR for RV in the SLE group was 23.61 (95% CI, 14.94–37.32). The results remained robust in the sensitivity analysis.

**Conclusion:** This nationwide population-based study revealed that SLE patients had a significantly higher risk of RV than non-SLE individuals.

## Introduction

Systemic lupus erythematosus (SLE) is an autoimmune-mediated diffuse connective tissue disease characterized by systemic autoimmune inflammation (1). SLE involves multiple organ systems with complex clinical manifestations and repeated disease courses. It often occurs in women of childbearing age (1–3). The etiology of SLE remains unclear, but genetic and environmental factors may play a role in the pathogenesis of the condition. A population-based study in Taiwan found that the average annual incidence of SLE was 4.87 per 100,000 persons between 2003 and 2008 (4). Additionally, an epidemiological study of SLE patients in the Thrace region of Turkey reported that the mean annual incidence of SLE was 4.44 per 100,000 persons, which was similar to the results of the Taiwanese study (5).

Retinal vasculitis (RV) is a type of inflammatory disease involving retinal blood vessels that is often accompanied by choroidal and vitreous inflammation. In severe cases, it can cause complications such as macular edema, which can significantly impair vision and even cause blindness. The pathogenesis of RV includes immune complex deposition and cellular immune response, causing a series of vascular damages and inflammatory responses (6). In addition to local retinal vascular inflammation, patients with RV can often have systemic vasculitis. The causes of RV are infectious and non-infectious, and most of the non-infectious causes are autoimmune diseases, including SLE.

Approximately 40% of SLE patients can have fundus changes (7). A previous cross-sectional study found that 2/69 (3%) of SLE patients had RV (6). RV can occur in the early or late stages of different SLE patients. Some patients have RV as the initial manifestation, and some patients develop severe RV when SLE disease progresses, even involving the optic nerve or causing retinal hemorrhage and severely impairing the patient's visual function (8–10). At present, compared with non-SLE individuals, the increase in the incidence of RV in SLE patients and the correlation between SLE and the risk of RV are unclear. Therefore, in this study, we explored the correlation between SLE and the risk of RV through a retrospective cohort study based on a population-based database.

## Patients and Methods

### Ethics Statement

This study was approved by the Institutional Review Board of Taichung Veterans General Hospital in Taiwan (approval number: CE17100B) and adhered to the principles of the Declaration of Helsinki. The requirement for informed consent was waived because of the retrospective nature of the study. The personal data of the study participants were rendered anonymous.

### Data Sources

The data used in this study were obtained from the 1997–2013 National Health Insurance Research Database (NHIRD) (11). In 1995, an obligatory National Health Insurance (NHI) was implemented in Taiwan. The Taiwan National Health Research Institutes (NHRI) collected and maintained the original claims data from the NHI administration and then released them to the NHIRD for research purposes. The claims data from the NHIRD consist of information on demographics, registration, residence, diagnosis, examinations, prescriptions, procedures, outpatient services, inpatient services, and medical expenditures. The NHIRD includes the stored medical claims for 99% of the 23.74 million Taiwanese residents (12). The Bureau of the NHI checks the original medical records regularly to improve the accuracy of claims data in the NHIRD. The study used multiple NHIRD datasets, including registration files, inpatient and outpatient files that provide diagnostic data based on the International Classification of Diseases, Ninth Revision, Clinical Modification (ICD-9-CM), clinical examinations, prescriptions, and medical expenses.

The BNHI set up a registry of catastrophic illness patients (RCIP). Patients with major or severe illnesses such as cancer, SLE, or rheumatoid arthritis were registered in the RCIP and were issued catastrophic illness certificates (CICs). A CIC is issued if at least two qualified specialists validate the diagnosis after a thorough review of the original medical records. All patients with CICs are exempted from copayment. The BNHI also randomly selected insured individuals who received medical services in 2000 to build a representative database of one million people (Longitudinal Health Insurance Database, LHID2000). We identified SLE patients from RICP who had CIC of SLE and selected non-SLE individuals from the LHID2000.

### Study Design

**Figure 1:**
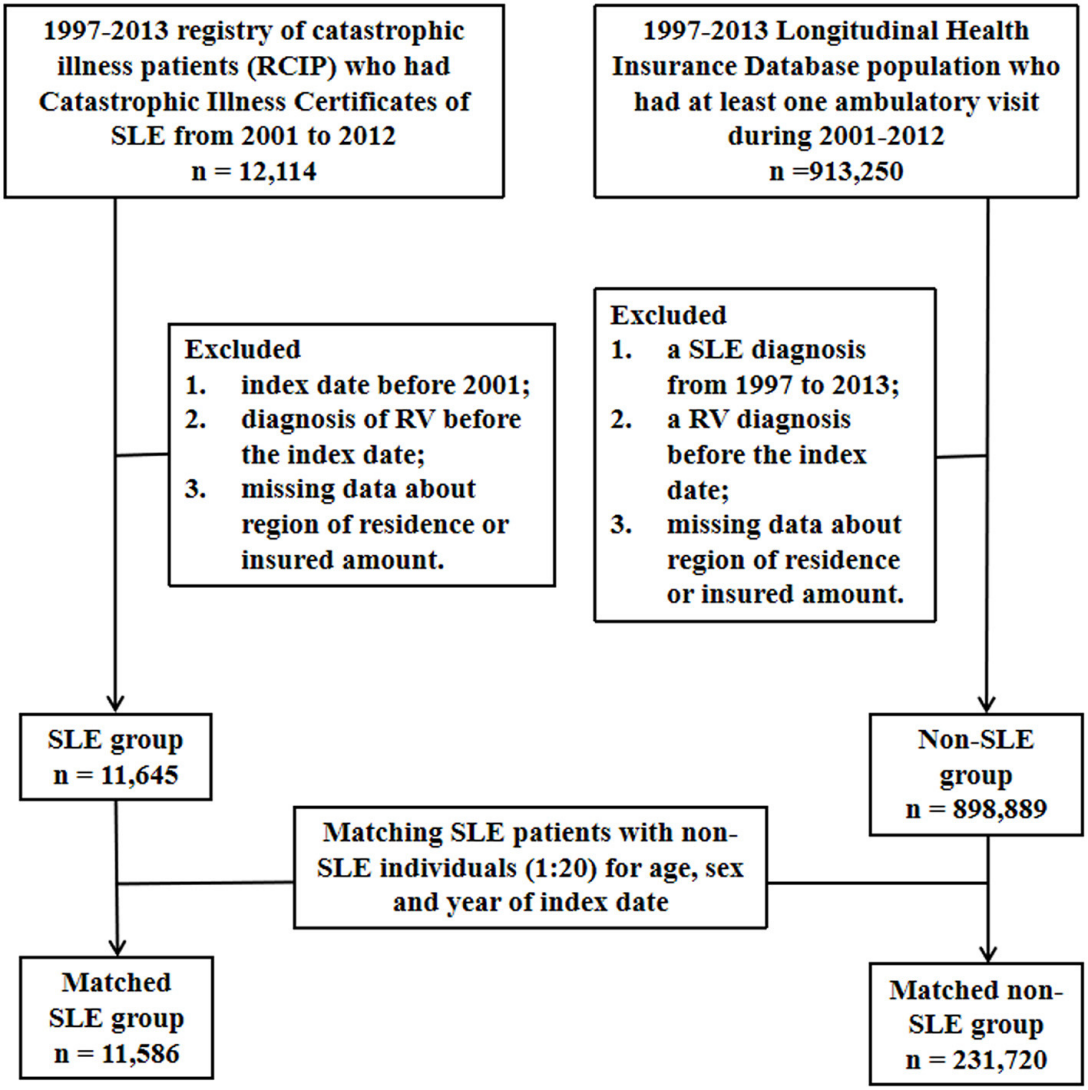
Flowchart of the study design. SLE, systemic lupus erythematosus; RV, retinal vasculitis.

This was a nationwide population-based matched cohort study. The flowchart of the patient enrolment procedure is shown in Figure 1.

### Patients With SLE From the Entire Population in Taiwan

A sample of the nationwide population from the period between 2001 and 2012 was selected from the NHID. The participants in this study were newly diagnosed with SLE (ICD-9-CM code 710.0) between 2001 and 2012 (*n* = 12,114). The index date was defined as the date on which SLE was first diagnosed. The exclusion criteria were as follows: (1) index date before 2001, (2) diagnosis of RV before the index date, and (3) missing data on the region of residence or the insured amount. In the end, a total of 11,645 SLE patients met the inclusion criteria.

### Non-SLE Individuals From the Representative One Million Sample of the Population in Taiwan

The non-SLE comparison group in this study was randomly selected from the representative one million sample of the population of LHID2000 who had at least one ambulatory visit during 2001–2012. The exclusion criteria were as follows: (1) a diagnosis of SLE from 1997 to 2013, (2) a diagnosis of RV before the index date, and (3) missing data regarding the region of residence or the insured amount. Ultimately, 898,889 individuals met the criteria for inclusion in the non-SLE group.

### Matched SLE Patients and Non-SLE Individuals

To examine the association between SLE and the risk of RV, we used individuals matching for sex, age, and year of the index date to select SLE patients and non-SLE individuals in a 1:20 ratio. Follow-up began on the index date and ended when the patient was diagnosed with RV, when the patient withdrew from the NHI due to any cause such as death or displacement, or when the study ended (December 31, 2013), whichever occurred first.

### Outcome

ICD-9-CM code 362.18 was used to identify RV patients. The main outcome of this study was time from the index date to the first date of RV diagnosis. To ensure the validity of RV definition, RV was defined in three different scenarios. First, a diagnosis of RV was made in at least one outpatient visit by a qualified ophthalmologist or in at least one admission with a diagnosis of RV. Second, only patients in scenario 1 who were treated with glucocorticoids, disease-modifying antirheumatic drugs (DMARDs) (methotrexate, sulfasalazine, leflunomide, and hydroxychloroquine), or immunosuppressants (cyclophosphamide, cyclosporin, azathioprine, and mycophenolate mofetil/mycophenolic acid) within 6 months after the first diagnosis of RV were considered to have RV. Third, only patients in scenario 1 who were treated with DMARDs or immunosuppressants within 6 months after the first diagnosis of RV were considered to have RV.

### Potential Confounders

Potential confounders included urbanization of the patient's residence, the level of the payroll-related insured amount as a proxy indicator of the participant's income, and selected comorbidities. The urbanization of the individual patient's residence was categorized into four clusters according to population density (people/km^2^), population ratio of elderly subjects aged >65 years, ratio of individuals with educational levels of college or above in the population, number of physicians/100,000 persons, and ratio of agricultural workers in the population (13). The payroll-related insured amount was used as a proxy indicator of the patient's income and was categorized into two levels based on the median value (i.e., low income, < the median value; high income, ≥the median value). Comorbidities that could be potential confounders of the association between SLE and RV risk included human immunodeficiency virus infection (ICD-9-CM codes 042–044, V08), diabetes mellitus (ICD-9-CM code 250), antiphospholipid antibody syndrome (ICD-9-CM code 289.8), hypertension (ICD-9-CM codes 401–405), cerebral vascular accident (ICD-9-CM codes 430–438), inflammatory bowel disease (ICD-9-CM codes 555–556), chronic liver diseases (ICD-9-CM codes 571 and 573), chronic kidney disease (ICD-9-CM code 585), rheumatoid arthritis (ICD-9-CM code 714.0), systemic sclerosis (ICD-9-CM code 710.1), Sjogren's syndrome (ICD-9-CM code 710.2), ankylosing spondylitis (ICD-9-CM code 720.0), Behcet disease (syndrome) (ICD-9-CM code 136.1), giant cell arteritis (ICD-9-CM code 446.5), polyarteritis nodosa (ICD-9-CM code 446.0), granulomatosis with polyangiitis (ICD-9-CM code 446.4), relapsing polychondritis (ICD-9-CM code 733.99), sarcoidosis (ICD-9-CM code 135), multiple sclerosis (ICD-9-CM code 340), Vogt–Koyanagi–Harada disease (ICD-9-CM code 364.24), toxoplasmosis (ICD-9-CM code 130), tuberculosis (ICD-9-CM codes 010–018), Whipple's disease (ICD-9-CM code 040.2), syphilis (ICD-9-CM code 090–097), lyme disease (ICD-9-CM code 088.81), cat scratch disease (ICD-9-CM code 078.3), chronic obstructive pulmonary disease (ICD-9-CM codes 490–496), hyperlipidemia (ICD-9-CM codes 272.0–272.4), asthma (ICD-9-CM code 493), hyperthyroidism (ICD-9-CM code 242), herpes simplex (ICD-9-CM code 054), herpes zoster (ICD-9-CM code 053), and cytomegalovirus infection (ICD-9-CM code 078.5). Comorbidities were identified if the corresponding ICD-9-CM codes were present in at least three ambulatory visits or at least one inpatient visit within 1 year before the index date.

### Statistical Analysis

We counted follow-up person-years and the number of persons diagnosed with RV, calculated the incidence of RV (cases per 100,000 person-years), and estimated the incidence rate ratio with its 95% confidence interval (CI). Multivariable Cox proportional hazard regression analysis was then used to estimate the adjusted HR (aHR) with 95% CI for RV. Three different models were used to investigate the effects of SLE on the risk of RV based on covariates: model 1, SLE alone; model 2, SLE and demographic variables (i.e., urbanization level of the patient's residence and insured amount according to the payroll); and model 3: SLE, demographic variables, and selected comorbidities.

Sensitivity analysis was used to estimate the risk of RV in patients with SLE exposure who were in age-matched and sex-matched populations under different RV definitions. Kaplan–Meier curves were generated on the cumulative incidence of RV in the SLE and non-SLE groups. The differences between the curves were evaluated using the log-rank test. In all of our studies, *p* < 0.05 was considered statistically significant. All statistical analyses were performed using the Statistical Analysis Software Version 9.4 (SAS Institute Inc., NC, USA).

## Results

The baseline characteristics of the SLE and non-SLE groups are shown in Table 1. We matched the two groups for sex, age, and year of the index date at a 1:20 ratio. Ultimately, there were 231,720 matched individuals in the non-SLE group and 11,586 patients in the SLE group (Figure 1). By comparing the urbanization and low income proportions of the two groups, we found that the proportion of suburban and rural patients in the SLE group and the proportion of low income patients were higher than those in the non-SLE group. The incidence of comorbidities in the SLE group was higher among the sex-matched and age-matched populations (Table 1). The mean follow-up periods in the SLE and non-SLE groups were 6.43 and 7.04 years, respectively.

**Table 1:** Baseline characteristics of the participants in the SLE and non-SLE groups.

	**1:20 age–sex matching**		
	**Non-SLE**	**SLE**	***P*** **-value**
	***n*** **= 231,720**	***n*** **= 11,586**	
Sex			1.000
Female	202,040 (87.2)	10,102 (87.2)	
Male	29,680 (12.8)	1,484 (12.8)	
Age(years)			1.000
<30	92,160 (39.8)	4,608 (39.8)	
30–45	70,600 (30.5)	3,530 (30.5)	
45–65	51,020 (22.0)	2,551 (22.0)	
≥65	17,940 (7.7)	897 (7.7)	
Urbanization			<0.001
Urban	74,124 (32.0)	3,491 (30.1)	
Suburban	110,336 (47.6)	5,536 (47.8)	
Rural	47,260 (20.4)	2,559 (22.1)	
Low income	117,260 (50.6)	6,066 (52.4)	<0.001
**Comorbidity^†^**		
Rheumatoid arthritis	422 (0.2)	767 (6.6)	<0.001
Sjogren's syndrome	205 (0.1)	1,014 (8.8)	<0.001
Antiphospholipid antibody syndrome	11 (0.005)	29 (0.3)	<0.001
Systemic sclerosis	10 (0.004)	129 (1.1)	<0.001
Ankylosing spondylitis	112 (0.05)	63 (0.5)	<0.001
Inflammatory bowel disease	165 (0.1)	25 (0.2)	<0.001
Behcet disease	17 (0.01)	13 (0.1)	<0.001
Giant cell arteritis	0 (0.0)	0 (0.0)	NA
Polyarteritis nodosa	1 (0.0004)	8 (0.1)	<0.001
Granulomatosis with polyangiitis	0 (0.0)	4 (0.03)	<0.001
Relapsing polychondritis	8 (0.003)	12 (0.1)	<0.001
Sarcoidosis	3 (0.001)	3 (0.03)	<0.001
Multiple sclerosis	5 (0.002)	11 (0.1)	<0.001
Vogt–Koyanagi–Harada disease	1 (0.0004)	1 (0.01)	0.003
Hypertension	16,128 (7.0)	1,539 (13.3)	<0.001
Diabetes mellitus	7,751 (3.3)	396 (3.4)	0.670
Hyperlipidemia	5,654 (2.4)	487 (4.2)	<0.001
Cerebral vascular accident	2,655 (1.1)	366 (3.2)	<0.001
Asthma	2,167 (0.9)	221 (1.9)	<0.001
Chronic obstructive pulmonary disease	4,505 (1.9)	594 (5.1)	<0.001
Chronic kidney disease	890 (0.4)	301 (2.6)	<0.001
Chronic liver disease	3,415 (1.5)	991 (8.6)	<0.001
Hyperthyroidism	1,090 (0.5)	209 (1.8)	<0.001
Human immunodeficiency virus	19 (0.008)	2 (0.02)	0.305
Toxoplasmosis	8 (0.003)	3 (0.03)	<0.001
Tuberculosis	263 (0.1)	148 (1.3)	<0.001
Whipple's disease	0 (0.0)	0 (0.0)	NA
Syphilis	38 (0.02)	20 (0.2)	<0.001
Lyme disease	0 (0.0)	2 (0.02)	<0.001
Cat scratch disease	1 (0.0004)	1 (0.01)	0.003
Herpes simplex	173 (0.1)	61 (0.5)	<0.001
Herpes zoster	422 (0.2)	175 (1.5)	<0.001
Cytomegalovirus infection	3 (0.001)	35 (0.3)	<0.001

† *Comorbidity was identified within 1 year before index date*.

*SLE, systemic lupus erythematosus; NA, not applicable*.

**Figure 2:**
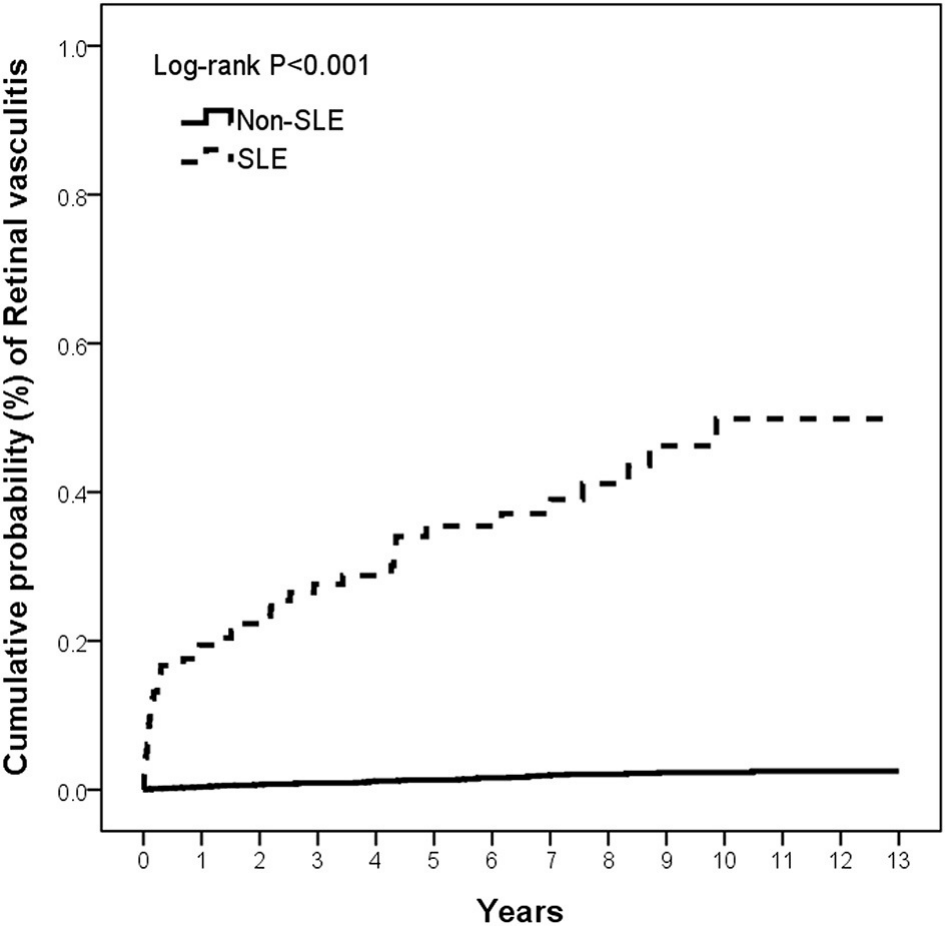
The cumulative incidence of retinal vasculitis in patients with and without SLE. SLE, systemic lupus erythematosus.

The cumulative incidence of RV was significantly higher in the SLE group than in the non-SLE group (*p* < 0.001) at the end of the follow-up period, and the Kaplan–Meier curves are shown in Figure 2. The incidence rates of RV were 56.39 per 100,000 person-years (95% CI, 56.33–56.44) and 2.45 per 100,000 person-years (95% CI, 2.45–2.45) in the SLE and non-SLE groups, respectively, and the incidence rate ratio of RV was 22.99 (95% CI, 14.91–35.45) (Table 2). The results revealed that there was an increased risk of RV in the SLE group.

**Table 2:** Incidence of retinal vasculitis in the SLE and non-SLE groups.

	**1:20 age–sex matching**	
	**Non-SLE**	**SLE**
*n*	231,720	11,586
Follow-up person-years	1,631,175	74,486
Retinal vasculitis, n (%)	40 (0.17)	42 (0.36)
Incidence rate^*^ (95% CI)	2.45 (2.45–2.45)	56.39 (56.33–56.44)

* *Incidence rate, cases per 100,000 person-years*.

*SLE, systemic lupus erythematosus; CI, confidence interval*.

The adjusted HRs for RV was estimated via Cox proportional hazard regressions, which are provided in Table 3. In all three models, SLE patients were found to have a significantly higher risk of developing RV with consistency. From model 1 to model 3, the HRs under SLE exposure were 22.43 (95% CI, 14.55–34.59), 22.67 (95% CI, 14.70–34.96), and 23.61 (95% CI, 14.94–37.32), respectively. Additionally, in all models, we did not find that differences in sex, age, urbanization, low income, and comorbidity affected the relative risk of RV.

**Table 3:** Cox proportional hazard regressions for estimation of adjusted HRs on retinal vasculitis.

	**1:20 age-matched and sex-matched population**		
	**Model 1: SLE exposure alone**	**Model 2: SLE exposure + demographic variables**	**Model 3: model 2 + medical utilization and comorbidities at baseline**
SLE	22.43 (14.55–34.59)	22.67 (14.70–34.96)	23.61 (14.94–37.32)
**Urbanization**			
Urban		Ref.	Ref.
Suburban		0.52 (0.32–0.84)	0.52 (0.32–0.84)
Rural		0.55 (0.30–1.01)	0.55 (0.30–1.01)
**Low income**		1.06 (0.68–1.63)	1.06 (0.69–1.64)
**Comorbidity^†^**			
Rheumatoid arthritis			1.54 (0.55–4.34)
Sjogren's syndrome		0.53 (0.13–2.23)	
Antiphospholipid antibody syndrome		Cannot estimate	
Systemic sclerosis		Cannot estimate	
Ankylosing spondylitis		Cannot estimate	
Inflammatory bowel disease		Cannot estimate	
Behcet disease		Cannot estimate	
Polyarteritis nodosa		Cannot estimate	
Granulomatosis with polyangiitis		Cannot estimate	
Relapsing polychondritis		Cannot estimate	
Sarcoidosis		Cannot estimate	
Multiple sclerosis		Cannot estimate	
Vogt–Koyanagi–Harada disease		Cannot estimate	
Hypertension		1.07 (0.48–2.41)	
Diabetes mellitus		1.94 (0.65–5.86)	
Hyperlipidemia		0.69 (0.16–3.05)	
Cerebral vascular accident		1.44 (0.34–6.19)	
Asthma		Cannot estimate	
Chronic obstructive pulmonary disease		2.06 (0.64–6.66)	
Chronic kidney disease		Cannot estimate	
Chronic liver disease		0.74 (0.23–2.38)	
Hyperthyroidism		1.07 (0.15–7.75)	
Human immunodeficiency virus		Cannot estimate	
Toxoplasmosis		Cannot estimate	
Tuberculosis		Cannot estimate	
Syphilis		Cannot estimate	
Lyme disease		Cannot estimate	
Cat scratch disease		Cannot estimate	
Herpes simplex		Cannot estimate	
Herpes zoster		1.54 (0.21–11.28)	
Cytomegalovirus infection		8.35 (1.13–61.75)	

† *Comorbidity was identified within 1 year before index date*.

*SLE, systemic lupus erythematosus; HR, hazard ratio*.

Table 4 shows the results of the RV risk estimation under SLE exposure using sensitivity analysis. Owing to the lack of relevant laboratory test results in the database, we divided the analysis into three scenarios based on different definitions of RV events. Detailed scenarios are shown in Table 4. The RV aHRs of SLE patients in scenarios 1–3 were 23.61 (95% CI, 14.94–37.32), 81.34 (95% CI, 42.05–157.34), and 434.78 (95% CI, 103.95–1,818.63), respectively. We found a high correlation between SLE exposure and severe RV requiring treatment with DMARDs or immunosuppressants. It also suggested that SLE patients were at markedly increased risk of RV development.

**Table 4:** Sensitivity analysis in the estimation of the retinal vasculitis risk for SLE exposure in age-matched and sex-matched populations.

**Scenario**	**Definition of retinal vasculitis event**	aHR^*^ (95% CI)
1	At least one outpatient visit with RV diagnosis made by a qualified ophthalmologist or 1 admission with RV diagnosis (main finding)	23.61 (14.94–37.32)
2	Scenario 1 + treated with systemic corticosteroids or DMARDs or immunosuppressants^†^	81.34 (42.05–157.34)
3	Scenario 1 + treated with DMARDs or immunosuppressants^†^	434.78 (103.95–1,818.63)

* *Adjusted variables included urbanization of residence, low income, and comorbidities listed in Table 1*.

† *The treatment of retinal vasculitis was identified within 6 months after first diagnosis of retinal vasculitis*.

*aHR, adjusted HR; SLE, systemic lupus erythematosus; DMARDs, disease-modifying antirheumatic drugs*.

## Discussion

To our knowledge, this is the first retrospective cohort study to use nationwide population-based data to assess the correlation between SLE and RV risk. In the current study, we found that patients with SLE had an increased risk of RV compared with individuals without SLE. Moreover, according to different Cox regression models, we concluded that all SLE patients had a high risk of RV. Better understanding of the correlation between SLE and RV could facilitate the early diagnosis of RV in SLE patients.

The ocular manifestations of SLE include skin diseases involving the eyelids, secondary Sjogren's syndrome, scleritis, RV, and neuro-ophthalmological diseases. Although ocular manifestations are not part of the SLE classification criteria, as many as one-third of SLE patients can be observed to have ocular manifestations (14). RV and SLE are both autoimmune diseases with a common immunological basis and produce a common immunological damaging effect. RV and SLE can occur concomitantly or alternately. There have been reports of a case with acute necrotizing RV as the primary manifestation of SLE (15) and a case of SLE patients with optic neuritis and RV as the main manifestations (16). The similarity between the cases was that the patients were all young women. In addition, studies have shown that the degree of fundus lesions is closely associated with the course of SLE, which can reflect the pathological status and degree of other organs (17). When SLE patients have central nervous system involvement, they are more likely to involve the eyes, causing active retinopathy, optic neuropathy, and retinal vascular occlusive diseases (7, 18). Therefore, it is recommended that clinicians perform regular fundus examinations on SLE patients to assess the patient's disease progression and treatment.

The mechanism of RV in patients with SLE has not been fully elucidated. It is currently believed that SLE can cause RV through a variety of mechanisms, including immune complex deposition and other antibody-related mechanisms, vasculitis, and thrombosis (19). RV and non-ocular systemic manifestations of SLE can occur concomitantly or alternately. Ushiyama et al. found that retinopathy was associated with anticardiolipin antibodies and central nervous system lupus (6). SLE patients with retinopathy had higher serum creatinine levels and maximum SLE disease activity index (SLEDAI) than those without retinopathy (6). In a prospective study of 550 SLE patients, Stafford-Brady et al. found that 88% of patients with active SLE were associated with lupus retinopathy, and retinopathy was a marker of poor survival in SLE patients (7). These studies all suggested that the presence of anticardiolipin antibodies, vasculitis, nervous system diseases, and urinary system diseases in SLE was associated with the occurrence of retinopathy, which was consistent with our research results. These antibodies and systemic diseases may be the reasons why SLE patients are more likely to develop RV.

This study had several upsides. First, the database that we used comprised nationwide longitudinal population-based data. Second, the study was supported by a large-scale sample to avoid selection and recall bias. This allowed our hypothesis to proceed smoothly, and the result can be considered more comprehensive and complete. However, this study also had several limitations. First, the NHID lacks data on some clinical characteristics, such as smoking habits, lifestyle, and family history, which may all be risk factors for the development of RV. Although we did undertake some measures to deal with some confounders, such as the diagnosis of chronic obstructive pulmonary disease due to a history of smoking, residual confounders may still have existed and increased the risk of bias. Second, because the data in the NHID are anonymous, we could not obtain laboratory data to confirm the diagnosis of patients, such as laboratory indices of blood and urine samples, fundus photograph results, fluorescein angiography, and imaging results. The laboratory data of some patients suggested viral infections such as CMV infection, but the doctor missed the diagnosis, which may have caused errors in our results. Nevertheless, we explored consistency in the results under different definitions of RV in the sensitivity analysis, and the cohort analysis of Taiwan NHID has also shown high levels of quality and validity in other studies (20, 21). Third, unless SLE patients have eye discomfort, rheumatologists rarely perform eye-related examinations, which results in many patients failing to receive timely RV diagnosis, causing errors in the results. In addition, the study lacks data from people from other countries and races, and whether the results can be extrapolated to other regions requires further investigation. Therefore, these limitations need to be addressed through further research to confirm our conclusions.

In conclusion, this nationwide population-based cohort study demonstrated that SLE patients are at a very high risk of incidental RV. Clinicians should provide appropriate monitoring and education for SLE patients for the risk of RV. If necessary, early intervention can be provided to reduce the risk of blindness and other disabilities. Further research is needed in the future to identify the possible mechanisms of these correlations.

## Data Availability Statement

The original contributions presented in the study are included in the article/supplementary material, further inquiries can be directed to the corresponding author/s.

## Ethics Statement

This study was approved by the Institutional Review Board of Taichung Veterans General Hospital in Taiwan (approval number: CE17100B). Written informed consent for participation was not required for this study in accordance with the national legislation and the institutional requirements.

## Author Contributions

X-HC and J-CS wrote the manuscript. JW and H-HC conducted bioinformatics analysis, analyzed the data, and drew diagrams. H-YM made a lot of contributions to the design of the research, conducted data analysis, graph generation, and wrote the manuscript. All authors read and approved the final manuscript.

## Funding

This work was supported by a grant from the National Natural Science Foundation of China Grants [81760298] and the 139 Program for the High-Level Medical Talents in Guangxi Province.

## Conflict of Interest

The authors declare that the research was conducted in the absence of any commercial or financial relationships that could be construed as a potential conflict of interest.

## Publisher's Note

All claims expressed in this article are solely those of the authors and do not necessarily represent those of their affiliated organizations, or those of the publisher, the editors and the reviewers. Any product that may be evaluated in this article, or claim that may be made by its manufacturer, is not guaranteed or endorsed by the publisher.
